# Safety and Efficacy of Corneal Cross-Linking in Patients Affected by Keratoconus: Long-Term Results

**DOI:** 10.3390/medsci11020043

**Published:** 2023-06-16

**Authors:** Karl Anders Knutsson, Paola Noemi Genovese, Giorgio Paganoni, Oriella Ambrosio, Giulio Ferrari, Arianna Zennato, Michela Caccia, Madeleine Cataldo, Paolo Rama

**Affiliations:** Cornea and Ocular Surface Unit, San Raffaele Scientific Institute, Via Olgettina 60, 20132 Milan, Italy

**Keywords:** corneal collagen cross-linking, keratoconus, corneal ectasia

## Abstract

The present study evaluated the effectiveness and safety of corneal collagen cross-linking (CXL). A total of 886 eyes with progressive keratoconus were enrolled in a retrospective cohort study in a tertiary care university hospital. CXL was performed using a standard epithelium-off Dresden protocol. Visual outcomes, maximum keratometry (Kmax), demarcation line measurements, and complications were recorded. Visual outcomes and keratometric data were analyzed in a subgroup comprising 610 eyes. Uncorrected distance visual acuity (UDVA) improved from 0.49 ± 0.38 LogMAR to 0.47 ± 0.39 LogMAR (*p* = 0.03, n = 610) three years after the procedure, while corrected distance visual acuity (CDVA) improved from 0.15 ± 0.14 LogMAR to 0.14 ± 0.15 LogMAR (*p* = 0.007, n = 610). A significant reduction of Kmax from 56.28 ± 6.10 to 54.98 ± 6.19 (*p* < 0.001, n = 610) was observed three years after CXL. In five eyes (0.82%, 5/610) keratoconus progression continued after CXL. Three eyes were retreated successfully with documented refractive and topographic stability after five years. In the 35 eyes that completed 10 years of follow-up, mean visual acuity and topographic parameters remained stable. In conclusion, CXL is a safe and effective treatment for avoiding keratoconus progression. Long-term data are encouraging, supporting a high safety profile for this procedure.

## 1. Introduction

Keratoconus is a noninflammatory and degenerative corneal ectasia, which usually involves both eyes and progresses over time [1]. Diagnosis is based on clinical features and corneal topography/tomography findings which reveal irregular astigmatism associated with reduced corneal thickness in most cases. In its first stages, keratoconus determines reduction of vision due to the development of myopia and irregular astigmatism. Eyeglasses can provide good visual acuity in the early stages of disease, until irregular astigmatism intensifies, requiring utilization of rigid contact lenses to obtain adequate results. In patients with progressive keratoconus, visual acuity with contact lenses may be reduced due to corneal opacities and corneal transplantation may become necessary. Corneal collagen cross-linking (CXL) involves a photochemical reaction which results in the creation of covalent bonds between collagen fibrils in the corneal stroma, resulting in increased corneal stiffness. Numerous studies have confirmed the efficacy of CXL in halting the progression of keratectasia in both the adult [2,3,4,5,6,7,8,9,10,11] and pediatric [1,12,13,14,15,16,17,18,19,20] population. Studies with long-term follow-ups of ten years have been recently reported [21,22,23]. The present study has the objective of adding evidence regarding the safety and long-term efficacy of CXL in patients affected by keratoconus.

## 2. Materials and Methods

The data of 1168 eyes of 886 consecutive patients with a diagnosis of keratoconus from June 2008 to September 2019, were retrospectively studied. The ethics committee of the hospital approved the study (San Raffaele Hospital), and informed consent was required by all patients. In cases of minors, parents signed the consent form. The study followed the ethical standards of the Declaration of Helsinki.

A diagnosis of keratoconus was formulated through different clinical and topographical findings typical of keratoconus. These included: patterns of keratoconus on topography, maximum keratometry (Kmax), simulated K values, and inferior–superior differences [24]. Disease progression was considered when Kmax increased by one or more diopter (D) in twelve months. Topography associated with complete ophthalmological evaluation and refraction was repeated every three months in patients under 14 years of age, and every six months in patients over the age of 14. Only patients with at least one year of follow-up were included. Patients with other forms of ectasia were excluded. Patients with reduced corneal thickness < 400 µm, corneal scars, history of keratitis of infectious nature, dry eye, corneal endothelial cell pathology, autoimmune pathology, or previous ocular surgery were excluded. If patients were wearing contact lenses, they would be asked to remove them three weeks prior to examinations.

Patients received a complete ophthalmological examination with the measurement of uncorrected distance visual acuity (UDVA), corrected distance visual acuity (CDVA), topographic examination with CSO-Eye Top Topographer and CSO-Sirius Topo-Tomography (CSO, Florence, Italy), preoperative and postoperative pachymetry, and specular biomicroscopy (Cellcheck-KONAN-Medical, Hyogo, Japan). All UDVA and CDVA values were recorded with Snellen charts and converted to LogMAR (Logarithm of the minimum angle resolution) for statistical analysis. Visual acuity measurements obtained with this method may be associated with a reduced precision and higher variance. The Eye-Top Topographer was used for patients receiving treatment before 2012, whereas Sirius topo-tomography was utilized for the subsequent patients. Each eye was measured with the same instrument at different time intervals. Baseline parameters were measured on the date of the surgical treatment. All topographic measurements were repeated three times, and the highest quality scan was selected and analyzed. Preoperative pachymetry was performed using an ultrasound probe by locating the thinnest point in a set of 5 measurements in proximity of the cone apex (Pachmate-DGH, Exton, PA, USA) in patients receiving treatment before 2011, thereafter corneal thickness was measured with Visante optical coherence tomography (OCT) (Zeiss-Meditec, Jena, Germany). Postoperative pachymetry was performed using the same preoperative method. The stromal demarcation line was identified through anterior segment OCT one month after CXL in all eyes (n = 886). A high resolution scan of the central cornea was processed at 180° orientation and the demarcation line was measured using a caliper tool function present in the manufacturer’s software. Patients returned to the clinic one day after treatment, at five days to remove the contact lens, at one month, six months, one year, and once a year thereafter for follow-up. Only one eye per patient was randomly selected for statistical purposes with a specific randomization software program. A total of 886 eyes were included in the safety analysis, whereas 610 eyes had completed at least three years of post-operative follow-up and were included in the visual outcome analysis.

### 2.1. Surgical Technique

CXL with riboflavin and ultra-violet A (UVA) light was performed in all eyes following a standard published protocol [8]. Topical anesthetic (lidocaine 4% or oxybuprocaine hydrochloride 0.2%) was utilized a few minutes prior to the procedure. The corneal epithelium was removed in the central 9 mm, using a blunt instrument and a 0.1%riboflavin-20%dextran solution (Ricrolin; Sooft, Montegiorgio, Italy) was instilled for 30 min (soaking phase). The central 9 mm of the cornea was then exposed to UVA radiation (370 nm) through a solid-state device (Vega X-Linker; CSO, Florence, Italy) at 3 mW/cm^2^ for 30 min (5.4 J/cm^2^ total energy), with an application of riboflavin every 150 s. If necessary, anesthetics were reapplied. Surgery was successful in all cases. A few pediatric patients were accompanied by a parent in the operating theater. The majority of patients received topical anesthesia alone. Postoperatively, levofloxacin 0.5% drops were instilled and a bandage contact lens was utilized for 5 days until complete epithelial healing. Patients received levofloxacin (0.5%) drops six times daily for five days, sodium hyaluronate lubricant drops 0.15% four times daily for thirty days, and fluorometholone 0.1% drops four times daily for three weeks following bandage contact lens removal.

### 2.2. Statistical Analysis

Statistical analysis was performed by entering the raw data into Microsoft Excel (Microsoft-Corp., Seattle, DC, USA, 2013 Version) and Statistical Package for Social Sciences (SPSS, Chicago, IL, USA, Version-20). The Shapiro–Wilk test was used to verify if data sets followed a normal distribution, in order to apply specific parametric or non-parametric tests. Vision parameters were compared using a Wilcoxon signed rank test, while keratometric values were compared using a paired *t*-test. Spearman correlation was utilized to determine if there was a correlation between demarcation line depth and Kmax. The statistical significance level was defined as a *p*-value < 0.05. To compensate for repeated measurement errors, we performed a Bonferroni correction, adjusting calculated *p*-values.

## 3. Results

A total of 1168 eyes of 886 patients were selected for this retrospective study. At the time of the study, not all patients had completed the same follow-up visits. Two subgroups were created to allow for more specific analyses; one group focusing on safety, and the second on visual outcomes. Only one eye per patient was randomly selected for statistical purposes. A total of 886 eyes were included in the safety analysis, whereas 610 eyes had completed at least three years of post-operative follow-up and were included in the visual outcome analysis. Data are reported as mean ± standard deviation. Mean age of the patients was 22.48 ± 6.72 years, with 682 male subjects and 204 female subjects (M:F ratio 3.3:1), and 131 patients (14.7%) were aged under 18, yielding an adult/minor ratio of 5.76. Mean pre-surgery pachymetry of the thinnest point was 472.67 ± 38.21 µm (n = 886); all patients had pachymetric values greater than 400 µm. In nine eyes, the procedure was performed under general anesthesia, while in the remaining cases, topical anesthesia alone was utilized. Table 1 summarizes the pertinent characteristics of the study patients.

**Figure 1:**
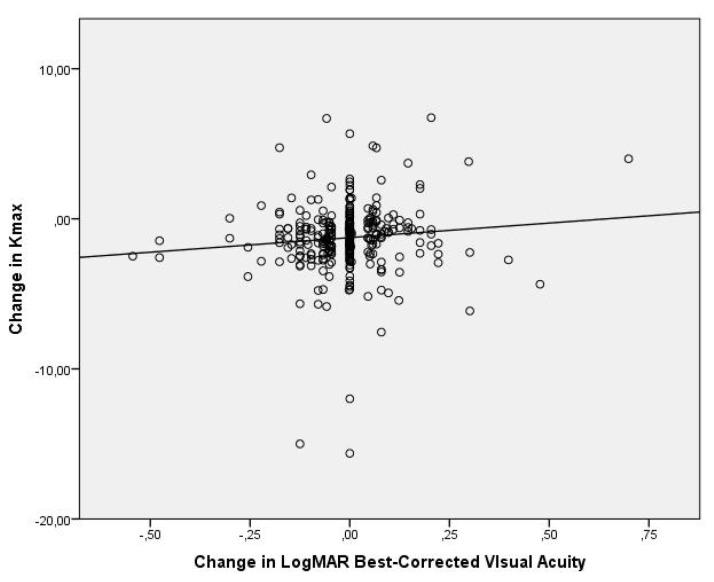
Scatter plot of the change in BCVA (LogMAR) versus the change in Kmax at 3 years postoperatively. The correlation coefficient is 1%.

**Figure 2:**
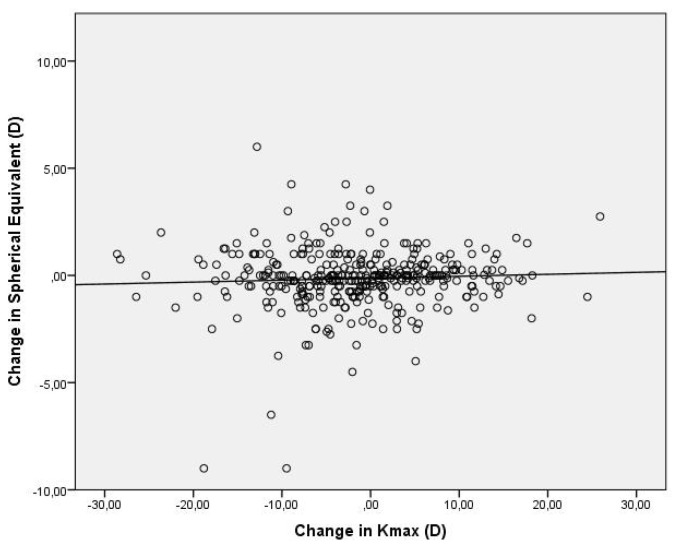
Scatter plot of the change in spherical equivalent versus the change in Kmax at 3 years postoperatively. The correlation coefficient is 0.3%.

Average UDVA improved from 0.49 ± 0.36 LogMAR to 0.47 ± 0.38 LogMAR three years after the procedure, while CDVA improved from 0.15 ± 0.15 LogMAR to 0.14 ± 0.15 LogMAR (n = 610). Changes were statistically significant with respective *p*-values *p* = 0.03 and *p* = 0.007. A significant decrease in Kmax from 56.28 ± 6.11 to 54.98 ± 6.18 (*p* < 0.001, n = 610) was observed after three years of follow-up. The relationship between corneal flattening (change in Kmax) and CDVA improvement (change in LogMAR) was weak (1% correlation), as shown in Figure 1. Furthermore, there was no significant relationship between the change in spherical equivalent and corneal flattening (0.3% correlation) as shown in Figure 2.

Pachymetry of the thinnest corneal point was 467.75 ± 37.38 µm (n = 610) at the baseline and 463.45 ± 40.44 µm (n = 610) at the three year follow-up (*p* = 0.32). The demarcation line of the CXL treatment was measured in all eyes one month after the procedure, and had a mean value of 254.24 ± 76.78 µm (n = 886). The demarcation line was clearly visible in most cases (94.8% 840/886) and relatively easy to trace in the less obvious cases. Demarcation line depth was not significantly correlated with a reduction in Kmax, a potential marker for efficacy of CXL. (Spearman‘s Rho = 0.21, *p* = 0.86, n = 610).

A total of 519 eyes had a complete follow-up at five years, whereas 35 eyes reached 10 years of follow-up. At five years, UDVA and CDVA remained stable compared to the three-year follow-up, 0.46 ± 0.36 LogMAR (*p* = 0.22) and 0.14 ± 0.18 LogMAR (*p* = 0.18), respectively. The results of patients with the full 10 year follow-up are summarized in Table 2. The data from the long-term follow-up demonstrate stability of visual acuity, except for a significant improvement at the 5 year follow-up. Kmax reduction compared to the baseline was confirmed at all time intervals, indicating corneal flattening which remained stable during the 10 year follow-up.

When considering eyes with a three year follow-up (n = 610), keratoconus remained stable without signs of progression at three years in 99.2% of cases receiving CXL treatment. Five eyes (0.82%) showed signs of keratoconus progression, which was defined as an increase in Kmax by more than 1 diopter after treatment. Two patients had documented progression, but were not retreated due to corneal thinning. Both patients were satisfied with their visual acuity; one patient was spectacle-independent, whereas the other used RGP contact lenses. Three patients with progression after initial CXL were retreated using the same protocol. Even though progression occurred and refractive astigmatism increased, CDVA remained stable before retreatment. All patients that were retreated showed clinical and topographic stability after at least five years of follow-up. The characteristics of patients with progression of keratoconus after CXL are summarized in Table 3. Of note, two of the five patients had suboptimal demarcation line depths of 153 µm and 174 µm. In some cases, ocular surface alterations, mainly corresponding to mild forms of dry eye, determined altered keratometry readings with fluctuating Kmax values. In these patients, lubricants were commenced, and topography was repeated one week later.

With regards to safety and complications, five patients were affected by a progression of keratoconus after CXL. Six patients who progressively became intolerant to contact lenses after the procedure and were unsatisfied with spectacle corrected vision underwent deep anterior lamellar keratoplasty with good outcomes. The latter was not considered as a complication, since contact lens intolerance occurred at least three years postoperatively in all cases, and was unlikely correlated to CXL. A very mild form of corneal trace haze was present in all cases, but resolved from 6 to 12 months after treatment and did not impact visual acuity, except in nine cases in which anterior stromal opacity developed. CDVA decreased after the procedure in 11 eyes (1.24%, 11/886). In nine cases (1.02%), this was due to the formation of anterior corneal stromal opacity and scarring, whereas in two atopic patients, visual acuity decreased in relation to ocular surface inflammation and epithelial irregularity. In all of these cases, there were no clear signs of keratoconus progression. Twelve eyes experienced continuous corneal flattening (1.97%, 12/610) defined as corneal flattening greater than three diopters, that persisted after three years of follow-up. Ten patients (1.13%, 10/886) were diagnosed with peripheral sterile corneal infiltrates at the one-week postoperative examination. Infiltrates resolved after removal of the contact lenses and therapy with topical steroids, yielding a full recovery in all patients without any cases of infectious keratitis. Mild signs of dry eye were present in 10 eyes (1.13% 10/886). All cases resolved with instillation of lubricants, with no cases of chronic dry eye. No cases of persistent epithelial defects or epithelial hypertrophy were encountered. Table 4 summarizes the complications in our cohort.

## 4. Discussion

CXL has modified the management of keratoconus, and has been proven effective in both adults and children. The standard Dresden protocol and accelerated protocols have shown encouraging results in both age groups. More recently, protocols such as the Custom Fast protocol have been developed [25]. This protocol uses software based on a mathematical model derived from the Lambert–Beer law in combination with the temporal law of rate of consumption of riboflavin, allowing efficient customization of the procedure [26,27,28]. In the present study, we report the long-term results of CXL on a large cohort of patients affected by keratoconus. As follow-up data was not homogenous, we divided patients into two groups. A total of 886 eyes had completed at least one year of follow-up and were included in the safety analysis, which was the primary objective of the study, whereas 610 eyes with at least 3 years of data were included in the visual outcome analysis.

The average age of the patients was 22.48 ± 6.72 years, 682 patients were male and 204 were female, with a male:female ratio of 3.3:1, in line with other studies [11,18] In our study, both uncorrected and corrected visual acuity minimally improved three years after CXL; however, this finding is of limited clinical significance. Kmax decreased significantly by a mean of 1.30 ± 1.49 D after three years (*p* < 0.001, n = 610) indicating flattening of the conical corneal shape after treatment. These results are in line with previous studies [1,3,4,7]. Pachymetry of the thinnest corneal point decreased from 467.75 ± 37.38 µm to 463.45 ± 40.44 µm after three years (*p* = 0.32, n = 610). Corneal thickness was measured using an ultrasound probe in 56 of the 610 eyes, comprising the refractive outcome subgroup, whereas the remaining eyes underwent OCT pachymetry. These findings are in line with other studies that describe a long-term stability of pachymetry [4,7,9], whereas in the early post-operative period, a slight reduction in thickness may be observed, likely related to the compacting effect on collagen fibers and epithelial remodeling [29,30]. Since pachymetry measurements were performed using different instruments, the significance of data deriving from the present study is limited. Demarcation line values were similar to those reported by other groups [31] and were not significantly correlated with reduction in Kmax (Spearman’s Rho = 0.21, *p* = 0.86), as reported by other studies [20,32]. The demarcation line depth was lower than average, but comparable to a previous study of our group, perhaps indicating excessive riboflavin imbibition during CXL [20].

Long term-follow up data with a complete 10 year follow-up was limited, and available for only 35 patients. Data analysis revealed stability of topographic parameters and mean visual acuity, with three patients (8.6%) showing signs of progression, which will be discussed in the next paragraphs. These promising findings are comparable to the results of previous studies with a 10 year follow-up, indicating a long-term effectiveness of CXL [21,22,23]. Newer data with ten year follow-ups will become available, and continue to add evidence regarding the long-term success of CXL in stopping progressive keratoconus.

Previous studies in different countries have reported infectious keratitis as a rare event, with three large retrospective series stating percentages of 0.17% (4/2350 eyes), 0.5% (11/2025 eyes), and 1.3% (7/532 eyes) [33,34,35]. In our series, no infections occurred. This may be determined by the temperate climate in our country, and to the immunosuppressive status of our cases: no subjects were under therapy with chronic topical steroids or oral immunosuppressive therapy. Furthermore, precise instructions were given to every patient, stressing the importance of following the correct postoperative therapeutical regimen, the perils of infectious keratitis, and risk factors related to inappropriate contact lens wear. In the work by Shetty et al., the four subjects with keratitis had associated risk factors such as vernal conjunctivitis and oral immunosuppressive agents [33]. Maharana et al. reported several cases (n = 7) of keratitis after accelerated CXL and highlighted that four (57.1%) of the cases had vernal keratoconjunctivitis [35]. Our patient population had 3% of cases with vernal conjunctivitis and atopy, but ocular surface inflammation was minimal, thus justifying the absence of postoperative keratitis. In some cases, prior to surgery, a short course of steroids was utilized to reduce ocular surface inflammation. No short-term surgical complications were observed, other than presence of sterile infiltrates and temporary dry eye. We reported sterile infiltrates in 10 eyes (1.13%), compatible with findings from other studies [34,35,36]. The presence of corneal haze differs greatly in different studies: different groups have noted rates of 9%, 9.1%, and 100% [4,7,34,37]. These important variances may be due to inter-observer differences, and may likely be underestimated because of limited impact on vision. Haze after CXL has diverse clinical characteristics compared to haze after other procedures, such as photorefractive keratectomy used for refractive surgery [38]. After CXL, a superficial change in the corneal stroma is observed, whereas after excimer laser ablation, it adopts a reticulated subepithelial pattern, suggesting that the mechanisms involved are likely different. The risk of haze after CXL has been reported to be greater in patients with lower pachymetry and steeper corneas [4]. We reported the presence of mild trace haze in all of our cohort, in line with the study by Wittig-Silva et al. [6] Haze disappeared from 6 to 12 months after treatment, and did not impact on visual acuity results. However, more specific tests, such as contrast sensitivity testing, were not performed, and could have been altered in the first months after CXL.

Eleven eyes (1.24%, 11/886) had a reduction of CDVA of one or two lines, which was a lower rate compared to data reported by Kymionis et al. and Koller et al. [37,39] In nine cases, a loss of visual acuity was related to the formation of anterior corneal stromal opacity and scarring (also defined as haze in other studies), whereas in two atopic patients, vision decreased due to inflammation and epithelial irregularity. In all of these cases, there were no clear signs of keratoconus progression. Anterior corneal scarring may be difficult to distinguish from corneal haze in some eyes, contributing to a potential underestimation of this complication.

Five eyes (0.82% 5/610) showed signs of keratoconus progression, which was defined as an increase in Kmax by more than one diopter after treatment. Two patients had documented progression, but were not retreated due to corneal pachymetry with a value < 400 µm. The patients are currently satisfied with their visual acuity, which has remained stable, and are being monitored. Three patients with progression after initial CXL were retreated using the same Dresden protocol technique, without intra- or post-operative complications. All patients that were retreated showed clinical and topographic stability after at least five years of follow-up. When analyzing the possible risk factors for progression in these five patients, we hypothesized that younger age could play a role. Three of the five patients were minors, and all patients were younger than the mean age of the study population. Furthermore, two patients had suboptimal demarcation lines (153 and 174 µm), which may be considered as an additional risk factor. The percentage of patients with keratoconus progression in our series was relatively low when compared to other studies [34,37,39,40,41,42,43]. Since our refractive analysis data regards patients with at least three years of follow-up, it is possible that the percentage of CXL failure is underestimated, and failure rates may increase with longer follow-up data. For example, when analyzing the relatively few patients that completed 10 years of follow-up, the rate of progression was greater (8.6%) but likely overestimated when considering that mean age was lower (19.09 ± 4.63), and the adult/minor ratio was lower compared to the initial population (2.09 vs. 5.76). Kmax is the most commonly used parameter to evaluate progression and CXL efficacy, but it has the main limitation of representing a small area of anterior curvature without taking into account thickness and posterior corneal measurements [44]. In our experience, Kmax values can fluctuate significantly after CXL, adding further confusion. Twelve eyes experienced continuous corneal flattening (1.97%, 12/610), which, in this study, was defined as corneal flattening greater than three diopters that persisted after three years of follow-up. The term “standard corneal flattening” has been used to indicate typical flattening which may occur in the first years after the procedure and tends to stabilize after three years [4,45]. However, in some cases, continuous corneal flattening may occur even after three years, and has been reported as a long-term complication of cross-linking with a prevalence of 6.3% [45]. We identified twelve eyes in which corneal flattening occurred after the three year time point. In all cases, Kmax values were greater than 55 diopters, which is a general risk factor for corneal flattening [6]. This percentage may be significantly affected by the retrospective study design, and only long-term prospective studies will reveal the actual rates of this late complication.

The principal weakness of the study is the lack of full data for all follow-up intervals, which potentially underestimates the number of complications. More than half of the patient population had completed three year post-operative examinations and were included in the visual outcome analysis. All patients (886 selected eyes) had completed at least one year of follow-up and were included in the safety analysis. This subgroup division was chosen because reported complications of CXL usually occur in the first weeks and months after treatment, with the exception of treatment failure and consequent progression, which can occur several years after the procedure. It is also important to highlight that progression after CXL is not readily identifiable, since, in the first years after the procedure, corneal remodeling takes place, and parameters used to assess progression may be temporarily modified. An additional limitation of the study is the variation in pachymetric measurements. In the first patients, most pachymetric measurements were performed with ultrasound, whereas in the later months, OCT was the most used technique as it was less invasive and more reproducible. Furthermore, even though each eye was measured with the same topographer, we compared eyes scanned with different instruments. This limitation could have an impact on keratometry data. To our knowledge, there are no studies evaluating the agreement between measurements of the two topographers used in this study. With regards to safety data, endothelial cell density measurements were not performed, even though it is important to monitor potential endothelial cell damage.

In conclusion, this large retrospective cohort study confirms the success and safety of CXL for the treatment of progressive keratoconus. Furthermore, this study adds important information regarding the long-term results contributing to the limited currently available literature.

## Figures and Tables

**Table 1:** Demographic and clinical characteristics of patients undergoing collagen cross-linking.

Number of eyes included	886
Age (years)	22.48 ± 6.72
Male:female ratio	3.3:1
Adult:minor ratio	5.76
Preoperative minimum corneal thickness (µm)	472.67 ± 38.21
Maximum keratometry (diopters)	56.33 ± 5.99
Uncorrected distance visual acuity (LogMAR)	0.50 ± 0.34
Corrected distance visual acuity (LogMAR)	0.15 ± 0.14

**Table 2:** Visual outcomes of 35 eyes with a full 10 year follow-up.

	UDVA	*p*	CDVA	*p*	Kmax	*p*
**Baseline (n = 35)**	0.72 ± 0.34	NA	0.19 ± 0.14	NA	59.49 ± 5.86	NA
**1 year**	0.66 ± 0.31	0.67	0.18 ± 0.12	0.63	58.32 ± 6.35	**˂0.001**
**3 years**	0.60 ± 0.29	0.22	0.15 ± 0.11	0.06	57.60 ± 5.99	**˂0.001**
**5 years**	0.60 ± 0.34	0.16	0.12 ± 0.12	**˂0.001**	56.67 ± 6.11	**˂0.001**
**10 years**	0.61 ± 0.38	0.30	0.16 ± 0.13	0.14	57.11 ± 6.22	**˂0.001**

Results are reported as mean ± standard deviation. UDVA: uncorrected distance visual acuity. CDVA: corrected distance visual acuity. Kmax: maximum keratometry.

**Table 3:** Patients with keratoconus progression after corneal collagen cross-linking.

Patient Number	Age	Sex	Demarcation Line Depth (µm)	Time of Onset of Progression (Years)	Retreatment
1	10	M	240	8	No. Good visual acuity with rigid gas permeable contact lens. Corneal thickness < 400 µm.
2	23	F	153	7	No. Good unaided visual acuity. Corneal thickness < 400 µm.
3	10	M	277	2	Yes. Repeated CXL after two years. Stable thereafter, follow-up 10 years.
4	19	M	174	2	Yes. Repeated CXL after two years. Stable thereafter, follow-up 5 years.
5	16	M	263	1	Yes. Repeated CXL after one year. Stable thereafter, follow-up 5 years.

**Table 4:** Postoperative complications.

Complication	N (Percentage %)
Treatment failure (progression)	5/610 (0.82%)
Mild trace haze	886/886 (100%)
Anterior stromal opacity (scarring)	9/886 (1.02%)
Reduction in best corrected visual acuity (one or two lines)	11/886 (1.24%)
Continuous corneal flattening	12/610 (1.97%)
Sterile infiltrates	10/886 (1.13%)
Temporary dry eye	10/886 (1.13%)

## Data Availability

The data related to this study is not publicly available due to privacy issues.
